# A novel and complex chromosomal variation in a child with developmental delay: A case report

**DOI:** 10.1097/MD.0000000000043092

**Published:** 2025-06-27

**Authors:** Hong Chang, Xiaohang Hu, Xinke Chen, Bin Chen

**Affiliations:** a Department of Medical Laboratory, Affiliated Hospital of Jining Medical University, Jining, Shandong Province, China.

**Keywords:** arrhythmia, chromosomal abnormality, developmental delay, hematuria, intellectual disability

## Abstract

**Rationale:**

Chromosomal variations generate diverse phenotypes, influenced by their size and genomic position. This report presents a previously unreported complex chromosomal rearrangement.

**Patient concerns:**

An 11-year-old Chinese boy presented with short stature and a decade-long history of growth delay.

**Diagnosis:**

The patient exhibited sinus tachycardia, arrhythmia, hematuria, developmental delay, intellectual disability, and reduced plasma growth hormone levels, alongside chromosomal abnormalities with associated deletions. G-banding analysis revealed a male karyotype (46, XY) with the following structural anomalies: r(1)(p13q32), t(6;21)(q21;q22), der(14)t(1;14)(p13;p12), and der(15)t(1;15)(q32;p12). copy number variation sequencing detected: del(1)(q31.3q32.1).seq[GRCh37/hg19](198,600,001–200,040,000) × 1, del(1)(q32.1).seq[GRCh37/hg19](200,960,001–202,480,000) × 1, del(6)(q14.1).seq[GRCh37/hg19](76,800,001–80,640,000) × 1. The patient’s phenotype was attributed to a complex chromosomal rearrangement involving 5 chromosomes, with partial deletions resulting from breakage and rejoining of chromosomes 1 and 6.

**Interventions:**

At age 3, the patient received rehabilitative therapy for developmental delay.

**Outcomes:**

No further treatment was provided following confirmation of the chromosomal abnormalities.

**Lessons:**

This case documents a novel chromosomal rearrangement for the first time, contributing valuable clinical insight and establishing a foundation for future research into related genetic disorders.

## 1. Introduction

Chromosomal variations (rare disorders caused by alterations in chromosome number or structure) can produce diverse clinical outcomes, including infertility, growth retardation, intellectual disability, and congenital syndromes.^[1–3]^ Complex chromosomal rearrangements involving 5 chromosomes have been reported in intellectual disability and malignancies; however, their molecular mechanisms and links to multisystem phenotypes remain poorly defined.^[4,5]^ These rare karyotypes, often comprising multiple structural variants such as insertional and reciprocal translocations with breakpoints in gene-rich regions, may significantly affect embryonic development and clinical presentation.^[6,7]^ Rearrangements involving 5 chromosomes with partial deletions from the breakage and rejoining of chromosomes 1 and 6 are particularly uncommon.

We describe an 11-year-old child with growth retardation and complex structural variations involving 5 chromosomes, including partial deletions from the breakage and reunion of chromosomes 1 and 6. The clinical features included intellectual disability, reduced growth hormone (GH) levels, sinus tachycardia, and hematuria.

## 2. Case report

In April 2022, an 11-year-old Chinese boy was admitted to our hospital for evaluation of growth retardation.

### 2.1. Medical history

The patient was born at term via uncomplicated vaginal delivery to non-consanguineous parents, with a birth weight of 4.1 kg. In infancy, he underwent surgical repair for a cleft lip and palate. Developmental milestones were delayed: teething at 15 months, independent walking at 2 years, and speech onset at 3 years. Early childhood was complicated by refractory diarrhea. At age 3, he received rehabilitation for intellectual disability. Over the past decade, he exhibited progressive growth delay, resulting in short stature.

### 2.2. Physical examination

At 11 years and 2 months, the patient measured 123.4 cm in height and weighed 23.0 kg. No facial dysmorphism or distinctive features were observed. Pulmonary auscultation was clear, with no rales or wheezes. Heart rate was 88 beats per minute. Testicular volume measured 1 mL bilaterally, with firm consistency. No spinal or limb deformities were noted; however, hyperextension of the finger joints with fixed distal interphalangeal joints was present.

### 2.3. Auxiliary examinations

Radiography of the left heel showed an unfused epiphysis, corresponding to a bone age of approximately 9 years. Pituitary magnetic resonance imaging revealed normal morphology and signal characteristics. Chest X-ray was unremarkable except for a calcified focus in the left axillary region. Ultrasonography of the left renal vein, heart, lungs, diaphragm, liver, biliary system, pancreas, spleen, kidneys, thyroid, and associated lymph nodes was normal.

Electrocardiography revealed sinus tachycardia with arrhythmia. Laboratory tests showed reduced plasma insulin-like growth factor 1 (IGF-1) at 55.7 ng/mL (reference: 69–316 ng/mL). An L-dopa stimulation test confirmed GH insufficiency, with a peak GH level of 3.781 ng/mL (reference > 10 ng/mL; Table 1). Urinalysis was positive for occult blood; urine microscopy identified 8 red blood cells per high-power field. All other laboratory findings (including complete blood count, stool analysis, liver and renal function, blood glucose, thyroid function, and cortisol levels) were within normal limits.

**Table 1:** Laboratory results of L-dopa growth hormone stimulation test.

Time after L-dopa injected (min)	0	30	60	90	120
The concentration of growth hormone (ng/mL)	0.379	0.374	3.781	1.652	0.278

**Figure 1.**
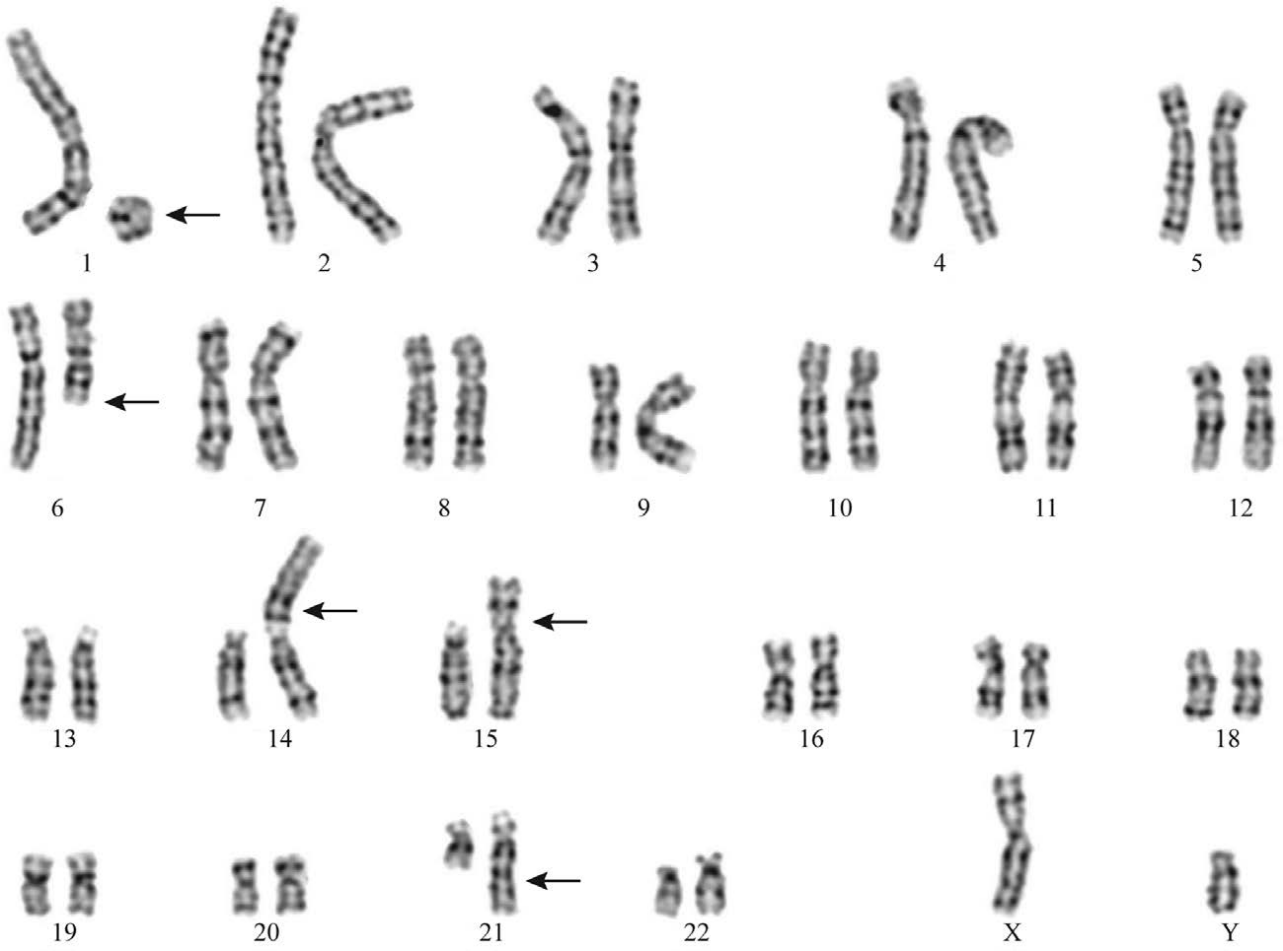
G-banded karyotype of the patient showing chromosomal abnormalities. Variant regions are indicated by black arrows.

**Figure 2.**
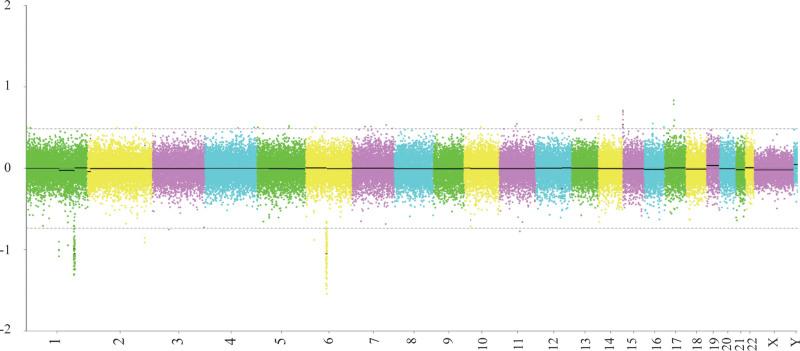
CNV analysis of the patient. The diagram displays detected CNVs. CNV = copy number variation.

**Figure 3.**
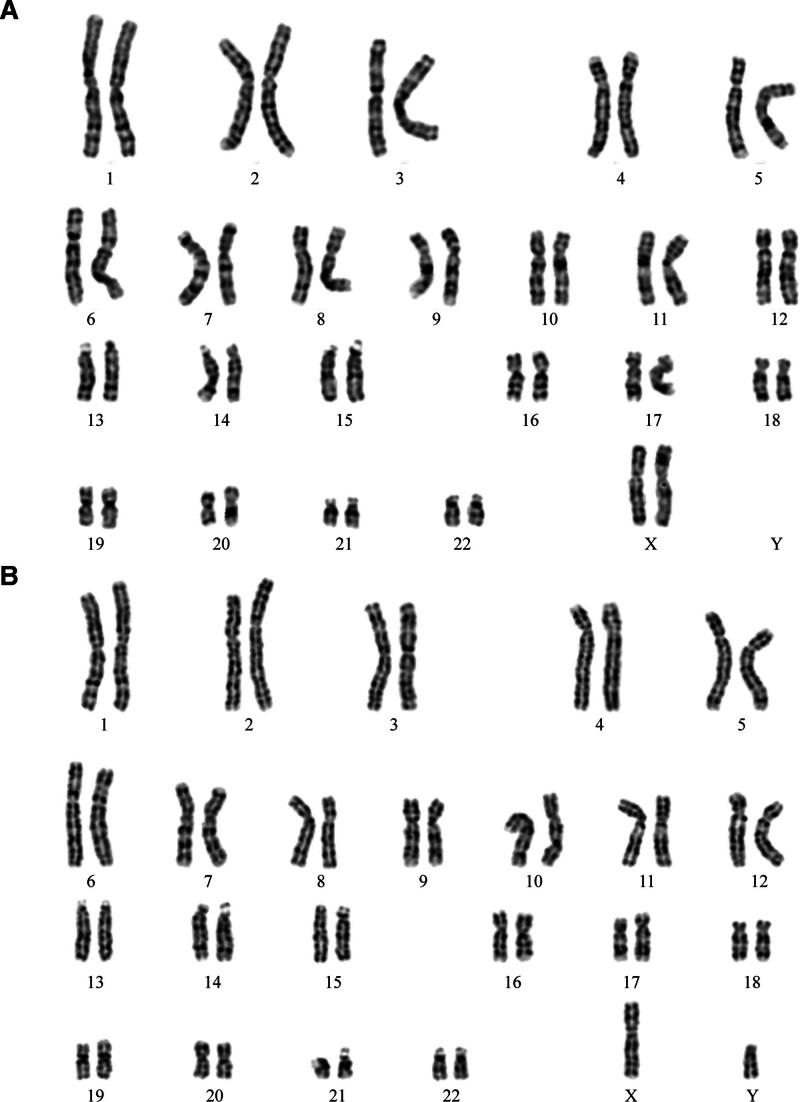
G-banded karyotypes of the patient’s parents. (A) Mother; (B) father.

G-banding analysis revealed a male karyotype (46, XY) with multiple chromosomal abnormalities: r(1)(p13q32), t(6;21)(q21;q22), der(14)t(1;14)(p13;p12), and der(15)t(1;15)(q32;p12). This karyotype, involving complex translocations across 5 chromosomes, was reviewed and confirmed as a novel finding by the expert panel of the *China Human Chromosome Abnormality Karyotype Database* (Fig. 1). To evaluate for balanced translocations, copy number variation sequencing (CNV-seq) was performed on peripheral blood, identifying the following deletions: del(1)(q31.3q32.1).seq[GRCh37/hg19](198,600,001–200,040,000) × 1, del(1)(q32.1).seq[GRCh37/hg19](200,960,001–202,480,000) × 1, del(6)(q14.1).seq[GRCh37/hg19](76,800,001–80,640,000) × 1 (Fig. 2). A 1.44 Mb deletion at 1q31.3–q32.1 encompassed 2 protein-coding genes (protein tyrosine phosphatase receptor, type C and nuclear receptor subfamily 5, group A, member 2) both listed in the Online Mendelian Inheritance in Man (OMIM) database. Based on a comprehensive review of Database of Genomic Variants, Clinical Genome Resource, Database of Genomic Variation and Phenotype in Humans using Ensembl Resources, ClinVar, and PubMed, this CNV was classified as of uncertain clinical significance. A 1.52 Mb deletion at 1q32.1 involved 25 protein-coding genes, including importin 9, leiomodin 1, and neuron navigator 1, all cataloged in OMIM. This CNV was similarly classified as of uncertain clinical significance. A 3.84 Mb deletion at 6q14.1 affected 8 protein-coding genes, including ELOVL fatty acid elongase 4, leber congenital amaurosis 5, and pleckstrin homology domain-interacting protein (PHIP), all listed in OMIM. No benign CNVs were documented for this region in Database of Genomic Variants. *PHIP* is recognized as haploinsufficient (HI score: 3) in Clinical Genome Resource, and loss-of-function variants are associated with Chung-Jansen syndrome (CHUJANS, OMIM #617991), characterized by developmental delay, intellectual disability, behavioral abnormalities, facial dysmorphism, and obesity. This CNV was classified as pathogenic. Whole-exome sequencing identified no additional clinically significant variants associated with the patient’s phenotype (intellectual disability, intractable diarrhea, dysphasia, cleft palate). Parental karyotyping was performed to assess inheritance of the chromosomal abnormalities; both parents exhibited normal karyotypes (Fig. 3A and B).

### 2.4. Follow-up and outcomes

The patient is currently enrolled in primary school. Compared with peers, he exhibits poor academic performance and short stature. He does not display obesity or the characteristic facial features of Chung–Jansen syndrome (fleshy earlobes, small nose, deep-set eyes, upturned upper lip, short smooth philtrum, or round face). Since diagnosis of developmental delay due to chromosomal rearrangement and deletions, the patient has not received targeted therapeutic interventions.

## 3. Discussion

We present a case of a child with sinus tachycardia, arrhythmia, hematuria, developmental delay, reduced plasma GH levels, and complex chromosomal abnormalities.

Pediatric growth retardation has a broad differential diagnosis, including intrauterine growth restriction,^[8]^ pituitary dwarfism,^[9]^ hypothyroidism, adrenal disorders, and chromosomal anomalies. In cases where parental karyotypes are normal, de novo chromosomal abnormalities may arise from gametogenic errors,^[10]^ environmental exposures (physical, chemical, or biological),^[11]^ pregnancy-related factors (e.g., teratogenic agents or adverse intrauterine conditions),^[12]^ or stochastic chromosomal mutations during early embryogenesis. In this patient, laboratory findings strongly implicate chromosomal abnormalities in the observed clinical phenotype. However, the precise etiology of the rearrangement remains unresolved.

Chromosomal abnormalities have been associated with a range of clinical features, including sinus tachycardia, arrhythmia,^[13]^ hematuria,^[14]^ growth retardation,^[15]^ and reduced plasma GH levels.^[16]^ In this case, a 3.84 Mb deletion at chromosome 6q14.1 (identified through clinical assessment and laboratory findings) was classified as a pathogenic CNV. This deleted region encompasses the PHIP gene, which encodes a substrate receptor in the ubiquitin ligase pathway and plays a key role in regulating cell cycle progression, survival, genomic stability, transcription, and embryonic development. *PHIP* mutations are linked to Chung–Jansen syndrome, a neurodevelopmental disorder characterized by global developmental delay, intellectual disability, dysmorphic facial features, and obesity.^[17]^ These mutations are typically de novo and exhibit overlapping yet variable phenotypes, primarily affecting neurological, craniofacial, somatic, and organ systems.^[18]^ The patient in this case presents with short stature, low body weight, mild intellectual impairment, and hyperextensible finger joints with immobile distal interphalangeal joints: features likely resulting from the *PHIP* deletion. Facial dysmorphism is notably absent. Such phenotypic variability is influenced by the type and location of the genetic alteration, inheritance pattern, and the individual’s broader genetic context. Here, the primary impact appears to be neurological, evidenced by intellectual developmental delay. Although the patient is enrolled in primary school following rehabilitation, academic performance remains limited. Physically, the phenotype is mainly expressed as short stature without craniofacial involvement. Functional abnormalities include arrhythmia and hematuria, suggesting organ-level effects. This case illustrates the considerable phenotypic variability associated with *PHIP* gene alterations and highlights the complexity of genotype–phenotype correlations in such disorders.

The patient in this case presents with 2 deletions on chromosome 1: a 1.44 Mb deletion spanning 1q31.3–q32.1 and a 1.52 Mb deletion within 1q32.1. Although both CNVs are classified as variants of uncertain clinical significance, they indicate a chromosomal break and segmental loss. However, CNV-seq has inherent limitations in accurately pinpointing breakpoints. For instance, although tools like CNVnator offer high sensitivity and a low false-discovery rate, their resolution for precise breakpoint detection remains limited. Breakpoint accuracy is highly dependent on sequencing depth and the choice of data analysis algorithm. Lower sequencing depth reduces detection precision, and differences among analytical tools can introduce further variability.^[19]^ In this case, the breakpoints are only 920 kilobases apart, making precise localization particularly challenging. Given current technical and bioinformatic constraints, inaccuracies in breakpoint identification due to limited resolution or sequencing depth cannot be ruled out. Although no pathogenic CNVs with identical characteristics are recorded in current databases, these deletions may still contribute to the patient’s clinical phenotype. Further research is needed to determine their potential pathogenic significance.

Laboratory tests showed reduced serum levels of GH and IGF-1, with GH deficiency confirmed by an L-dopa stimulation test. These findings may be related to the PHIP gene deletion, as PHIP regulates insulin sensitivity and glucose metabolism via insulin receptor substrate-1, potentially affecting GH and IGF-1 secretion and contributing to impaired growth.^[20]^ Microscopic hematuria may be associated with the LMOD1 gene, which encodes a smooth muscle cytoskeletal protein involved in maintaining renal vascular integrity.^[21]^ Additionally, the chromosomal translocation t(6;21) and derivative chromosome der(15) may disrupt *KCNE1*, a cardiac ion channel gene, offering a potential explanation for the observed sinus tachycardia and arrhythmia.^[22]^ While these gene–phenotype associations are biologically plausible, current evidence is insufficient to establish definitive causal links. The proposed connections remain speculative, primarily based on phenotypic features reported in related syndromes.

## 4. Conclusions

This case illustrates a rare instance of growth retardation in a Chinese patient with complex chromosomal abnormalities, presenting with sinus tachycardia, arrhythmia, hematuria, developmental delay, and low GH levels. Although CNV-seq has inherent limitations in breakpoint resolution, future advancements in molecular diagnostics and database resources may clarify these genotype–phenotype correlations. At present, the patient’s clinical features are most likely attributable to unbalanced chromosomal variation.

## Author contributions

**Data curation:** Hong Chang, Xiaohang Hu.

**Funding acquisition:** Xinke Chen, Bin Chen.

**Investigation:** Hong Chang.

**Writing – original draft:** Hong Chang, Bin Chen.

**Writing – review & editing:** Xiaohang Hu, Xinke Chen, Bin Chen.
